# Medications and Alcohol Craving

**Published:** 1999

**Authors:** Robert M. Swift

**Affiliations:** Robert M. Swift, M.D., Ph.D., is an associate professor in the Department of Psychiatry and Human Behavior at Brown University and associate chief of staff for research and education at the Providence Veterans Administration Medical Center in Providence, Rhode Island

**Keywords:** AOD (alcohol and other drug) craving, anti alcohol craving agents, alcohol withdrawal agents, drug therapy, neurobiological theory, alcohol cue, disulfiram, naltrexone, calcium acetylhomotaurinate, dopamine, serotonin uptake inhibitors, buspirone, treatment outcome, reinforcement, neurotransmitters, patient assessment, literature review

## Abstract

The use of medications as an adjunct to alcoholism treatment is based on the premise that craving and other manifestations of alcoholism are mediated by neurobiological mechanisms. Three of the four medications approved in the United States or Europe for treating alcoholism are reported to reduce craving; these include naltrexone (ReVia^™^), acamprosate, and tiapride. The remaining medication, disulfiram (Antabuse^®^), may also possess some anticraving activity. Additional medications that have been investigated include ritanserin, which has not been shown to decrease craving or drinking levels in humans, and ondansetron, which shows promise for treating early onset alcoholics, who generally respond poorly to psychosocial treatment alone. Use of anticraving medications in combination (e.g., naltrexone plus acamprosate) may enhance their effectiveness. Future studies should address such issues as optimal dosing regimens and the development of strategies to enhance patient compliance.

Criteria for defining alcoholism vary widely. Most definitions of alcohol dependence include the following descriptors: a compulsion to seek and consume alcohol, a loss of control over consumption after beginning a drinking session, and a strong likelihood of relapse during or after withdrawal.^1^ These manifestations may be accompanied by a conscious desire or urge to consume alcohol (i.e., craving). Craving can occur spontaneously, or it can be elicited by internal or external stimuli, known as cues (see sidebar by Tiffany on p. 216 ). Internal cues may include emotional states (e.g., anxiety) or symptoms of acute alcohol withdrawal. External cues may include exposure to alcohol-related environments or objects (e.g., bottles of alcoholic beverages or advertisements).

Results of craving research are often difficult to interpret, because the subjective nature of craving makes it difficult to assess and quantify. Also, the quality and intensity of craving may vary according to personal characteristics as well as environmental circumstances or experimental conditions. Nevertheless, phenomena associated with craving may have important implications for preventing and treating alcoholism. For example, high levels of craving are associated with increased probability of relapse, particularly during the early stages of the posttreatment period (Anton et al. 1996). In addition, treatments that reduce craving have been shown to reduce subsequent alcohol use (Monti et al. 1993).^2^

In the past decade there has been increasing interest in the use of medications (i.e., pharmacotherapy) to improve the effectiveness of psychosocial alcoholism treatment (Litten et al. 1996; Swift 1999). Alcoholism pharmacotherapy is based on the premise that alcohol use is mediated through specific neurobiological and behavioral mechanisms that initiate and maintain drinking. Several medications have demonstrated their ability to reduce alcohol consumption in humans, and several of these drugs are reported to reduce alcohol craving. Researchers can reasonably conclude that medications which reduce craving may be effective in alcoholism treatment.

## Assessing and Measuring Craving

Because craving is a subjective phenomenon, researchers commonly assess its intensity based on the self-reports of study participants.^3^ The simplest craving assessments are single-item questionnaires. In the most basic approach, the subject responds to a question (such as, “How much do you desire alcohol right now?”) by selecting one of three to five statements that indicate increasing levels of intensity (e.g., from “not at all” to “very much”). In some studies the patient rates his or her craving on a visual analog scale (VAS). In this approach, the patient places a mark on a line that is approximately 4 inches long and divided into arbitrary units beginning with zero. The reliability of single-item craving assessments is variable.

Multi-item questionnaires include the Obsessive Compulsive Drinking Scale (Anton et al. 1995), which contains 14 questions, each rated on a scale from 0 to 4, and the Alcohol Urge Questionnaire, which contains 8 statements rated from 1 to 7 (Bohn et al. 1995).

Craving is sometimes assessed by measuring certain physiological changes thought to accompany craving, such as changes in heart rate, blood pressure, salivation, and sweat gland activity. Finally, craving can be assessed by directly observing a subject’s drinking behavior. Behavioral measurements may include number of drinks consumed, time elapsed between cue exposure and initiation of drinking (i.e., latency), and time elapsed between commencement and completion of drinking.

## The Neurobiology of Craving

Research during the past decade has expanded our knowledge of the brain mechanisms that mediate the use of alcohol and other drugs (AODs). Alcohol may be consumed for its rewarding effects, which are sometimes consciously perceived as a pleasurable state (e.g., the effects of mild alcohol intoxication), or as relief from a painful or uncomfortable state (e.g., amelioration of withdrawal symptoms). Repeated exposure to a rewarding stimulus increases the probability that a person will continue to seek, approach, and maintain contact with the particular stimulus. Stated simply, a person who finds alcohol use rewarding may subsequently drink more often and in larger quantities. This process is referred to as positive reinforcement when its goal is to attain a pleasurable state and is referred to as negative reinforcement when its goal is to ameliorate an unpleasant state (Roberts and Koob 1997).^4^

AODs may activate the same neurobiological mechanisms that respond to conventional reinforcers, such as food and sexual activity (Di Chiara et al. 1996). Reinforcement of AOD use is associated with release of the neurotransmitter dopamine in a brain region called the nucleus accumbens (Roberts and Koob 1997) (see textbox above). Long-term exposure to alcohol can lead to compulsive use that characterizes dependence (Roberts and Koob 1997).

**The Role of Neurons and Neurotransmitters**

The ends of adjacent nerve cells, or neurons, are generally separated from one another by microscopic gaps called synapses. Most neurons communicate with one another by releasing chemicals called neurotransmitters, which cross the synapse and attach to a receptor protein on the receiving neuron. Each neurotransmitter binds preferentially to a single family of receptor subtypes, each of which may then stimulate, inhibit, or modulate a specific physiological function. A single neuron generally releases only one or a few types of neurotransmitters but may contain several types of receptors. A neurotransmitter released into a synapse is usually quickly removed by chemical degradation or by transporter molecules that carry the neurotransmitter back into the neuron that released it. The function of a neurotransmitter can be increased or mimicked by drugs, medications, or other chemical agents (i.e., agonists) or decreased, inhibited, or reversed by other agents (i.e., antagonists).

Following repeated administration of alcohol, the brain attempts to restore normal function through physiological adjustments (i.e., counteradaptations) that tend to reduce alcohol’s initial sedating effects (Roberts and Koob 1997). When a person terminates a prolonged drinking session, these counteradaptations are unopposed, resulting in signs and symptoms of acute withdrawal. During this period, craving may represent a desire to self-medicate the anxiety and hyperexcitability that accompany this state.

Certain manifestations of counter-adaptation, presumably involving persistent dopamine dysregulation in the nucleus accumbens, can prolong vulnerability to craving long after the acute symptoms of withdrawal have subsided. This mechanism may account for the ability of cues, such as the sight, sound, or smell of alcohol, to trigger relapse years after an alcoholic has stopped drinking (Roberts and Koob 1997).

The processes involved in addiction include complex interactions among several neurotransmitters in addition to dopamine (see table, p. 209). For example, opioid peptides may mediate some of alcohol’s rewarding effects (e.g., euphoria), and serotonin may help regulate overall motivational and appetitive behaviors. In addition, both of the above-mentioned neurotransmitters influence dopamine activity in the nucleus accumbens. These interactions are further regulated by a balance between excitatory neurotransmitters (e.g., glutamate) and inhibitory neurotransmitters (e.g., gamma-aminobutyric acid [GABA]).

## Medications to Treat Alcohol Dependence and Craving

Four medications are currently marketed for treating alcohol dependence. Two of them—disulfiram (Antabuse^®^) and naltrexone (ReVia^™^)—have been approved for this purpose by the Food and Drug Administration in the United States. The two other medications—acamprosate (Geerlings et al. 1997) and tiapride—are used in various European countries, although these drugs have not been approved for use in the United States. Additional medications are used empirically by clinicians to treat alcohol dependence, and several other agents are being developed.

### Disulfiram

Disulfiram interferes with the metabolism of alcohol by the liver, permitting a toxic breakdown product of alcohol to accumulate in the bloodstream. Alcohol consumption following disulfiram treatment results in unpleasant symptoms, such as flushing, palpitations, difficulty breathing, headache, and nausea. Recent reviews of placebo-controlled clinical trials with disulfiram have failed to confirm the drug’s efficacy in alcoholism treatment (Hughes and Cook 1997). As an adjunct to psychosocial therapy, disulfiram may decrease the quantity (Chick et al. 1992) and frequency (Chick et al. 1992; Fuller et al. 1986) of drinking among recovering alcoholics, but the medication does not appear to increase the proportion of patients who maintain total abstinence (Hughes and Cook 1997). Disulfiram’s effects on craving have not been specifically evaluated. However, disulfiram has been shown to interfere with the metabolism of dopamine (Rogers et al. 1979), potentially influencing the development of craving.

### Opioid Antagonists

Because opioid peptides stimulate dopamine release in the nucleus accumbens, medications that block opioid activity may block the reinforcing effects of alcohol. Naltrexone, an opioid antagonist used (under the brand name Trexan^®^) to treat heroin addiction, has been approved for treating alcoholism.

**Table:** Presumed Major Functions of Some Specific Neurotransmitters Involved in Alcohol-Related Behaviors and Alcoholism

Neurotransmitter	General Function	Alcohol-Related Function
Dopamine	Regulates motivation, reinforcement, and fine movement coordination	Mediates reinforcement of alcohol consumption
Serotonin	Regulates bodily rhythms, appetite, sexual behavior, emotional states, sleep, attention, and motivation	May influence alcohol consumption, intoxication, and development of tolerance through 5HT_1_ receptors; may contribute to withdrawal symptoms and reinforcement through 5HT_2_ receptors; and may modulate dopamine release through 5HT_3_ receptors, thereby increasing alcohol’s rewarding effects (see dopamine)
Gamma–aminobutyric acid (GABA)	Serves as the primary inhibitory neurotransmitter in the brain	May contribute to intoxication and sedation; inhibition of GABA function following drinking cessation may contribute to acute withdrawal symptoms
Glutumate	Serves as the major excitatory neurotransmitter in the brain	May contribute to acute withdrawal symptoms; inhibition of glutamate function following drinking cessation may contribute to intoxication and sedation
Opioid peptides (including beta-endorphin)	Regulates various functions as well as produces morphine-like effects, including pain relief and mood elevation	Contributes to reinforcement of alcohol consumption, possibly through interaction with dopamine

NOTE: The effects of the extensive and complex interactions among these neurotransmitters are not fully understood.

In a randomized, placebo-controlled clinical trial (Volpicelli et al. 1992), patients who received 50 milligrams (mg) of naltrexone per day^5^ reduced both their drinking and craving. In the study, naltrexone was administered to patients participating in an alcoholism rehabilitation program that included group therapy, individual counseling, health education, and recreation. Craving was assessed weekly by means of a single-item, 10-point scale with 0 indicating no craving and 9 indicating “severe” craving. Although overall craving was low, averaging about three out of nine for the placebo group, the craving reported by the subjects taking naltrexone was lower over the entire 12 weeks of the study. Of the patients taking naltrexone, 23 percent relapsed, compared with 54 percent of the patients taking the placebo.

In a double-blind study of 97 alcoholics who were administered either a placebo or 50 mg of naltrexone daily for 12 weeks, the naltrexone-treated group had increased abstinence rates as well as decreased drinking and decreased reports of craving (O’Malley et al. 1992). All participants received psychosocial treatment consisting of either individual coping-skills/relapse-prevention therapy or supportive therapy without a specific coping component. Coping-skills therapy attempts to teach patients to identify and handle situations that place them at high risk for relapse to drinking. This approach includes instruction in self-monitoring practices; rehearsal of anger and stress management techniques; training in social, problem-solving, and decisionmaking skills; and development of alternative, abstinence-oriented leisure activities.

In this clinical trial (O’Malley et al. 1992), self-reported craving over the previous week was rated on a 20-point analog scale. An interaction existed between craving and the specific psychotherapy received. Abstinence rates were highest among participants receiving naltrexone in combination with supportive psychotherapy. Among patients who sampled alcohol during treatment, however, those who received naltrexone and coping-skills therapy were least likely to relapse. A subsequent analysis of data from this study (Jaffe et al. 1996) indicated that naltrexone was most effective in reducing drinking in patients who reported high levels of craving at the time they entered the study.

Laboratory investigations with naltrexone generally support the clinical findings of reduced craving in naltrexone-treated subjects. In recently abstinent alcoholic patients who received either 50 mg of naltrexone daily or a placebo for 7 days, patients who received naltrexone were less likely to report cue-induced craving than patients who received the placebo. Among the subjects who reported craving, however, the absolute *intensity* of craving had not decreased from levels measured at the start of the experiment. Craving was measured on a seven-point analog scale (Monti et al. 1999).

Another study of naltrexone’s effect on laboratory-induced craving in recently detoxified, alcohol-dependent subjects found that a single 50 mg dose of naltrexone reduced craving in response to alcohol cues but not in response to a sweetened control beverage (Rohsenow 1998). In this study, craving was assessed as the urge to drink as rated on a seven-point scale.

Not all studies on naltrexone have shown reductions in craving. One study conducted in persons who used both alcohol and cocaine found no effects of naltrexone in reducing alcohol and cocaine craving. However, in this study, levels of induced alcohol craving were low (Modesto-Lowe et al. 1997). In addition, a laboratory study that investigated the effects of four doses of naltrexone (ranging from 0 to 100 mg) on alcohol-dependent subjects found no effect on the subjects’ urge to drink (Farren et al. 1999).

Some researchers have suggested that craving can be assessed by determining the time latency between presentation and consumption of a drink, with shorter latency indicating increased desire to consume alcohol. Two such experiments using this approach were conducted in which nonalcoholic drinkers consumed alcohol under observation in a public bar. In the first experiment, subjects were randomly assigned to receive either naltrexone or a placebo for 8 consecutive days under double-blind conditions prior to each of three 2-hour drinking sessions. The three sessions were separated by 2 to 3 weeks and occurred on the evening in which the last dose of naltrexone or the placebo had been administered. The experiment used a crossover design, in which subjects switched from naltrexone to the placebo or from the placebo to naltrexone prior to each subsequent drinking session. The results showed significant increases in “latency to sip” between the first and second alcoholic drinks, although no differences were found in the subjects’ self-reports on their urge to drink. A significant reduction in total alcohol intake also was observed during the naltrexone treatment compared with the placebo “treatment” (Davidson et al. 1996).

In the second experiment, 51 heavy beer drinkers were pretreated with either a placebo or 50 mg of naltrexone daily, each for 7 consecutive days prior to the drinking sessions. Again, the subjects’ latency to drinking increased during the naltrexone period compared with the placebo. The subjects who received naltrexone also consumed less alcohol, and the time they took to finish one drink was increased. In this experiment, subjects reported less of an urge to drink when they received naltrexone than when they received a placebo (Davidson et al. 1999).

In a randomized, double-blind, placebo-controlled study, Volpicelli and colleagues (1997) found no reduction in craving measured on a 10-point scale among subjects receiving naltrexone. However, these researchers have noted that many subjects dropped out of treatment prematurely and that the clinical effectiveness of naltrexone might be improved by techniques for enhancing medication compliance.

In summary, the data suggest that naltrexone’s beneficial effects in reducing alcohol consumption and increasing abstinence may be associated with the ability of the drug to block craving, including both the urge to drink and associated physiological responses.

### Dopamine Antagonists

Dopamine’s role in reinforcement suggests the possibility of using dopamine antagonists to reduce alcohol consumption and craving. In a double-blind, placebo-controlled laboratory study, 16 subjects diagnosed with either alcohol abuse or alcohol dependence reported less craving for alcohol and consumed less of their preferred alcoholic beverage after receiving the dopamine antagonist haloperidol (Haldol^®^), a medication commonly prescribed to treat severe psychiatric illness (Modell et al. 1993).

The dopamine antagonist tiapride, marketed in Europe for the treatment of alcohol abuse and dependence, was shown to be effective in increasing abstinence in a randomized, double-blind trial with more than 100 subjects conducted in Great Britain, although craving for alcohol was not specifically assessed (Shaw et al. 1994).

A preliminary, double-blind, placebo-controlled laboratory study of the recently approved antipsychotic medication olanzepine (Zyprexa^®^) on alcohol-induced stimulation and cue-induced craving found that pretreatment with olanzepine attenuated measures of alcohol- and cue-induced craving (Hutchison et al. 1998). Unlike traditional antipsychotic dopamine antagonists, this medication is less likely to produce neurological side effects, such as movement disorders.

### Serotonergic Medications

Several pharmacological agents that alter serotonergic function in the brain appear to reduce ethanol consumption in animals (Kranzler and Anton 1994; LeMarquand et al. 1994). However, in the clinical treatment of alcohol-dependent patients, the effects of most serotonergic medications seem to be only modest in reducing alcohol consumption. Such medications increase the overall activity of serotonin in synapses as well as agonists and antagonists at various serotonin-receptor subtypes.

#### Selective Serotonin Reuptake Inhibitors (SSRIs)

These medications, which appear to reduce alcohol consumption in animals, augment overall serotonergic function by interfering with the removal of serotonin from the synapse after its release (see textbox, p. 208). Some SSRIs are commonly prescribed for certain psychiatric disorders, such as depression and anxiety. Several human studies on heavy drinkers found SSRIs to reduce overall alcohol consumption by approximately 15 to 20 percent (Naranjo et al. 1994). In one study of 18 heavy drinkers that was conducted in a bar, the SSRI citalopram (Celexa^®^) reduced both drinking and self-reported craving for alcohol. However, studies with alcohol-dependent patients have yielded less impressive results (Kranzler et al. 1995). While some alcohol-dependent subjects treated with SSRIs have reported decreased craving and liking for alcohol, the reductions tend to be transient. Kabel and Petty (1996) report no differences in drinking outcome among severe alcoholics (mean = 19 drinks per day) who were treated with either fluoxetine (Prozac^®^) or a placebo, although craving decreased significantly in fluoxetine-treated subjects after 12 weeks.

#### Buspirone

Several serotonergic agonists and antagonists were observed to reduce alcohol consumption in animals and were tested in alcohol-dependent humans. Buspirone (Buspar^®^), which is prescribed for anxiety, demonstrated incomplete agonistic activity at a specific serotonin-receptor subtype (i.e., the 5HT_1_ receptor).

The observation that buspirone reduces alcohol consumption in alcohol-drinking rats led to human studies with nonanxious alcohol-dependent patients. In a study of 50 alcoholic subjects in treatment, Bruno (1989) found reduced drinking, less craving, and improved social and psychological status in patients receiving buspirone.

Several controlled clinical studies using buspirone have been conducted on patients with co-occurring alcoholism and anxiety disorders. In one such study, Tollefson and colleagues (1992) found that buspirone reduced patients’ anxiety levels, number of days desiring alcohol, and intensity of craving. However, other controlled clinical studies using buspirone have been negative. Malcolm and colleagues (1992) concluded that although buspirone combined with psychosocial treatment may help ameliorate psychiatric symptoms, the drug’s effects on alcohol consumption are limited.

#### Ritanserin

An antagonist of serotonin 5HT_2_ receptors, ritanserin may reduce alcohol consumption in rats (Litten et al. 1996). Human studies, however, are disappointing—for example, ritanserin was reported to reduce craving for alcohol in heavy social drinkers, although it did not decrease consumption (Litten et al. 1996).

The effectiveness of ritanserin for treating alcoholism was recently tested in a rigorous multicenter, placebo-controlled clinical trial in which 423 alcoholics received up to 5 mg of ritanserin per day concurrently with cognitive-behavioral therapy (Johnson et al. 1996). All study participants significantly reduced their alcohol consumption by the end of the 12-week study period. However, no difference in either craving or drinking level was observed between the patients who had received ritanserin and the patients who had received the placebo.

#### Ondansetron

Through its action at the 5HT_3_ receptor, serotonin helps regulate the release of dopamine into the nucleus accumbens, thereby affecting the development of reinforcement. Researchers have shown 5HT_3_-receptor antagonists to block dopamine release in animals and also to reduce alcohol consumption in rats (Litten et al. 1996). In a placebo-controlled outpatient trial with 71 socially stable, mildly alcohol-dependent men treated with the 5HT_3_ antagonist ondansetron (Zofran^®^), researchers found that a low dose, but not a high dose (0.25 versus 2.0 mg twice daily), of ondansetron moderately reduced alcohol consumption. A significant difference between treatment effects was observed only after excluding the heaviest drinkers (Sellers et al. 1994). In a human laboratory study, ondansetron reduced subjective measures of the desire to drink alcohol measured by VAS 1 hour after administration of the medication and also reduced some of the pleasurable effects of alcohol consumption (Johnson et al. 1993).

### Acamprosate

The medication acamprosate helps restore the balance of excitatory and inhibitory neurotransmission in the nucleus accumbens (Litten et al. 1996). The drug has been found to reduce alcohol consumption in rats trained to drink alcohol (Litten et al. 1996). In randomized, placebo-controlled clinical trials with human alcoholics, acamprosate significantly increased the proportion of patients who remained continuously abstinent as well as the duration of abstinence. Effects on craving for alcohol, however, have been more variable.

A 12-month multicenter study conducted in France compared the effects of 1.2 and 2 grams of acamprosate daily with that of a placebo (Paille et al. 1995). While the higher acamprosate dose significantly increased the proportion of subjects who remained abstinent at 6 and 12 months, no effect on alcohol craving occurred at these time periods. However, the higher dose of acamprosate reduced craving at 3 months, when the intensity of craving was much higher (Paille et al. 1995).

Additional randomized, double-blind, placebo-controlled clinical studies confirm that acamprosate increases the rate and duration of abstinence in alcoholics who receive concurrent psychosocial therapy, but all studies have been negative with respect to craving, as measured with single-item VAS. In a study using 600 subjects, Lhuintre and colleagues (1990) found more marked reductions in craving among the most severe alcoholics (i.e., those with the highest initial craving scores) but with no difference between the drug and the placebo groups. In a study by Sass and colleagues (1996), abstinence rates improved in treated alcoholics during a 48-week period. However, self-reported craving by VAS showed wide variability over this period and did not correlate with drinking behavior (Sass et al. 1996).

Acamprosate has been approved in several European countries for the treatment of alcohol dependence (see Geerlings et al. 1997) and is currently undergoing clinical testing in the United States (Litten et al. 1996).

### Sedative Medications

The sedative gamma-hydroxybutyrate is a neurotransmitter related to GABA and was formerly administered clinically as both a hypnotic and an anesthetic. In addition, the drug may effectively relieve some alcohol-withdrawal symptoms (Litten et al. 1996). In an open, multicenter trial with abstinent alcoholics, this medication reduced alcohol consumption and significantly reduced craving measured by the 14-item Alcohol Craving Scale (Addolorato et al. 1996). However, some subjects became dependent on the medication itself and began to take increasingly higher doses. Therefore, the drug should be administered to alcoholics only with extreme caution and close monitoring.

Excessive activity of the neurotransmitter norepinephrine has been implicated in some symptoms of withdrawal, and medications that inhibit norepinephrine may help alleviate withdrawal (Litten et al. 1996). Several medications that reduce norepinephrine-mediated arousal are reported to attenuate craving. Beta blockers (e.g., propranolol [Inderal^®^]) are a class of norepinephrine antagonists, many of which are prescribed to treat high blood pressure and symptoms of acute withdrawal symptoms (Gottlieb et al. 1994). A random, double-blind, placebo-controlled study (Gottlieb et al. 1994) also found beta blockers to be effective in reducing alcohol craving during withdrawal and early abstinence (to 1 year) as measured on a five-point scale. Alcoholics with high initial craving scores showed the greatest reduction, and completion of the program correlated with improvement in both the drug and the placebo groups. However, overall study completion rates were low; thus, no conclusions could be drawn about long-term relapse (Gottlieb et al. 1994).

## Summary

The concept of craving, although difficult to describe, is nevertheless important to alcoholism treatment research. Several medications that reduce human alcohol consumption in laboratory studies and clinical trials also reduce craving for alcohol. However, craving is not always associated with alcohol drinking, and a direct relationship does not always exist between medication-induced reductions in craving and reductions in drinking. A better understanding of the concept of craving and its relationship to alcohol drinking may lead to a better understanding of the neurobiology of alcohol dependence and subsequently to more effective clinical interventions.

Future studies should address such issues as optimal dosing regimens, matching patients with appropriate medications, and developing strategies to enhance patient compliance. In assessing craving, more reliance should be placed on experimentally validated, multi-item questionnaires, such as the Obsessive Compulsive Drinking Scale. Ongoing research on the effectiveness of prescribing medications in combination (e.g., naltrexone and acamprosate) may usher in a new era of alcoholism pharmacotherapy.

## Footnotes

1 Symptoms of acute withdrawal (e.g., tremors, agitation, and seizures) may occur following cessation or reduction of heavy drinking.

2 Craving alone is neither necessary nor sufficient for relapse.

3 This section presents a brief overview. For more detail, see the article in this issue by Drobes and Thomas, pp. 179–186.

4 Research suggests that in some alcoholics an aversive state may result from inherited deficiencies in serotonin or other neurotransmitters; this state may initially be alleviated by self-medication with alcohol but will actually worsen from long-term, continued consumption (Roberts and Koob 1997).

5 Fifty mg/day is standard dose of naltrexone for clinical treatment of opioid dependence (Volpicelli et al. 1992).
